# Reduction of Hydrogen Peroxide Accumulation and Toxicity by a Catalase from *Mycoplasma iowae*

**DOI:** 10.1371/journal.pone.0105188

**Published:** 2014-08-15

**Authors:** Rachel E. Pritchard, Alexandre J. Prassinos, John D. Osborne, Ziv Raviv, Mitchell F. Balish

**Affiliations:** 1 Department of Microbiology, Miami University, Oxford, Ohio, United States of America; 2 Center for Clinical and Translational Science, University of Alabama at Birmingham, Birmingham, Alabama, United States of America; 3 Department of Veterinary Preventative Medicine, The Ohio State University, Columbus, Ohio, United States of America; The University of Melbourne, Australia

## Abstract

*Mycoplasma iowae* is a well-established avian pathogen that can infect and damage many sites throughout the body. One potential mediator of cellular damage by mycoplasmas is the production of H_2_O_2_ via a glycerol catabolic pathway whose genes are widespread amongst many mycoplasma species. Previous sequencing of *M. iowae* serovar I strain 695 revealed the presence of not only genes for H_2_O_2_ production through glycerol catabolism but also the first documented mycoplasma gene for catalase, which degrades H_2_O_2_. To test the activity of *M. iowae* catalase in degrading H_2_O_2_, we studied catalase activity and H_2_O_2_ accumulation by both *M. iowae* serovar K strain DK-CPA, whose genome we sequenced, and strains of the H_2_O_2_-producing species *Mycoplasma gallisepticum* engineered to produce *M. iowae* catalase by transformation with the *M. iowae* putative catalase gene, *katE*. H_2_O_2_-mediated virulence by *M. iowae* serovar K and catalase-producing *M. gallisepticum* transformants were also analyzed using a *Caenorhabditis elegans* toxicity assay, which has never previously been used in conjunction with mycoplasmas. We found that *M. iowae katE* encodes an active catalase that, when expressed in *M. gallisepticum*, reduces both the amount of H_2_O_2_ produced and the amount of damage to *C. elegans* in the presence of glycerol. Therefore, the correlation between the presence of glycerol catabolism genes and the use of H_2_O_2_ as a virulence factor by mycoplasmas might not be absolute.

## Introduction

*Mycoplasma iowae* is an economically important avian mycoplasma primarily associated with turkeys but also occasionally found in chickens [1]. Naturally occurring infections in turkeys commonly result in late embryo mortality, leading to a 2–5% decrease in hatchability, and leg abnormalities in offspring [2]–[4]. Experimental infections give rise to airsacculitis, stunting, poor feathering, and/or leg and joint problems [3], [4]. *M. iowae* can be recovered from the respiratory tract, gastrointestinal tract, spleen, and kidneys of orally infected day-old poults, suggesting invasiveness [5]. Atypically among cultured mycoplasmas, *M. iowae* exhibits a predilection for the intestinal tract. Yolk sac inoculation of eight-day old turkey embryos results in intestinal binding and colonization [6]. Oral inoculation of day-old poults also results in intestinal colonization with no signs of disease, with mycoplasmas isolated more frequently from the intestinal walls than its contents, for at least 21 d after inoculation [5].

Phylogenetically, *M. iowae* is in the *Mycoplasma muris* cluster within the pneumoniae group, with *Mycoplasma penetrans* as its best-characterized relative [7]. *M. iowae* has a terminal organelle with an internal cytoskeleton; this organelle attaches to host cells and is the leading cell pole during gliding motility [6], [8]. *M. iowae* is one of two known avian mycoplasmas, along with *Mycoplasma lipofaciens*, that can obtain energy from catabolism of both glucose and arginine [9], [10]. Despite considerable understanding of disease pathology resulting from *M. iowae* infection, as well as some knowledge about its cellular biology and biochemistry, the virulence factors of *M. iowae* are unidentified.

A draft genome sequence for *M. iowae* serovar I strain 695 [11] suggests some potential primary virulence factors, including two copies of genes for proteins related to an ADP-ribosylating and vacuolating toxin characterized in *Mycoplasma pneumoniae* [12] as well as genes for production of H_2_O_2_ through glycerol catabolism, a pathway that is widespread among mycoplasmas. In this pathway glycerol is imported into the cell via either the glycerol facilitator GlpF or an ABC transporter, phosphorylated by GlpK, and finally oxidized to dihydroxyacetone phosphate by GlpO (also known as GlpD in some species), using O_2_ and resulting in production of H_2_O_2_ [13], [14]. The H_2_O_2_ generated by GlpO can cause cellular damage either directly or through altering host gene expression [15]. H_2_O_2_ has been described as a virulence factor for several pathogenic mycoplasmas, including *Mycoplasma mycoides* subsp. *mycoides, Mycoplasma pneumoniae*, and *Mycoplasma pulmonis*. Onset time of pneumonia caused by *M. pulmonis* infection is shortened in mice lacking catalase activity, implicating H_2_O_2_ [16]. A *glpD* mutant of *M. pneumoniae* produces less H_2_O_2_ and causes less cytotoxicity than wild-type cells [14]. An *M. mycoides* strain that lacks components of the glycerol ABC transporter produces less H_2_O_2_ and causes disease with delayed onset and decreased severity in experimentally infected cattle as compared with a strain that has all the transport [17]–[19]. Several enzymes have been implicated in reversing H_2_O_2_-mediated damage to mycoplasma cells [20], [21], but direct degradation of H_2_O_2_ has not been described for these organisms.

It is unclear how well the existence of the glycerol catabolic pathway correlates with actual use of H_2_O_2_ as a virulence factor by mycoplasmas. The *M. iowae* serovar I genome sequence indicates the presence of *glpF*, *glpO*, and *glpK* [11], which could be taken to support a role for H_2_O_2_ in virulence of this organism. Thus far uniquely among mycoplasmas, it also contains a gene encoding a putative catalase, an enzyme that degrades H_2_O_2_, potentially interfering with H_2_O_2_-mediated virulence. In this report, we present the genome sequence of *M. iowae* serovar K strain DK-CPA, which also contains both the glycerol catabolism genes and the catalase gene, which we designate *katE*. The activity of the *katE* gene product as a catalase was tested by assaying for catalase activity in both *M. iowae* serovar K and *katE* transformants of *Mycoplasma gallisepticum*, a prolific H_2_O_2_ producer. All strains were also analyzed for the impact of *M. iowae* catalase on H_2_O_2_ production and resulting virulence via *Caenorhabditis elegans* toxicity assays modified for use with mycoplasmas. Our results, which constitute the first account of an active catalase in mycoplasmas, allow us to address the question of H_2_O_2_ as an *M. iowae* virulence factor.

## Materials and Methods

### Bacterial strains and growth conditions

Strains used in this study include *M. iowae* serovar K strain DK-CPA, *Mycoplasma genitalium* strain G37, *M. gallisepticum* strain R_low_, and *M. gallisepticum* transformants 55A–C and 56 A and C. All strains were grown at 37°C in 175-cm^2^ tissue culture flasks containing 50 mL SP-4 media [22] to mid-log phase (orange color). Transformants 55A-C and 56 A and C were grown in the presence of 4 µg mL^−1^ tetracycline. *Escherichia coli* strain DH5α was used for cloning and was grown in Luria-Bertani broth in the presence of 100 µg mL^−1^ ampicillin or 4 µg mL^−1^ tetracycline.

### Genome sequencing and analysis

Genomic DNA from *M. iowae* serovar K was sequenced at the Ohio State University Plant-Microbe Genomics Facility using the GS FLX system (454 Life Sciences). It was prepared with the GS FLX Titanium Rapid Library Preparation Kit (Roche) and sequenced using GS FLX Titanium Sequencing Kit XLR70. Shotgun sequencing data were assembled with the GS *De Novo* Assembler version 2.5.3 (Roche). Annotation was performed as previously described [23]. The draft genome project has been deposited at DDBJ/EMBL/GenBank under accession number AWQU00000000. The draft genomes of serovars K and I were compared using wgVISTA [24].

### Catalase sequence analysis

The predicted amino acid sequence of *M. iowae* serovar K catalase was subjected to PSI-BLAST [25]. Matches reported as having e values of 0 were aligned by CLUSTALX 2.1 [26] using default parameters and the phylogram was inspected using NJplot [27]. Sequences that clustered separately from the group that included *M. iowae* catalase were removed and the remaining sequences were again subjected to alignment and tree generation. Secondary structure was predicted using SOPMA [28].

### Detection of catalase activity

Cells from mid-log phase cultures of *M. iowae*, *M. gallisepticum*, and *M. gallisepticum* transformants were collected by centrifugation at 20,000×*g* for 20 min and washed three times with cold phosphate-buffered saline (PBS). Cells were smeared onto a clean microscope slide and one drop of 3% H_2_O_2_ was added. Catalase activity was indicated by the generation of bubbles.

To analyze H_2_O_2_ degradation, *M. iowae* and *M. genitalium* were grown in 24-well plate wells containing 1 mL of SP-4 media supplemented with 3% glycerol. Upon reaching mid-log phase, 1 mL of SP-4 containing 3% glycerol or mid-log phase *M. iowae* culture was added to *M. genitalium* culture and the amount of H_2_O_2_ remaining was measured over time using colorimetric test strips (EM Quant, range 0.5–25 mg L^−1^). Statistical analysis of results was calculated using repeated measures ANOVA and unpaired Student's T-test. Results represent three biological replicates with one technical replicate each.

### Construction of plasmids and transformation with *M. iowae katE*

To amplify *M. iowae katE* with a *Sal*I cloning site with or without the addition of a C-terminal 6xHis tag, primers upstream (5′-ATCGGTCGACAAATGCTGCAACAGCTGCAC-3′) and downstream (5′-ATCGGTCGACTAAACACAAAATTTGATTTAATC-3′ or 5′-ATCGGTCGACTTAATGATGGTGATGGTGGTGACCATATGCGTTTAATGGCAAGGT-3′, respectively) were synthesized (Fisher Scientific) and used for PCR with LongAmp Polymerase (NEB). Following addition of A overhangs using *Taq* polymerase (NEB), PCR products were ligated into linearized plasmid pCR2.1 (Invitrogen) and transformed into chemically competent *E. coli* cells. Sequencing at the Miami University Center for Bioinformatics and Functional Genomics confirmed 100% sequence identity to *M. iowae katE*. The resulting plasmids were named pOO54 and pOO53, respectively. These plasmids were digested with *Sal*I and the resulting 1.8-kb DNA fragments were ligated together with pTF20 [29] that had been linearized using *Sal*I. The resulting plasmids were named pOO56 and pOO55, respectively.

To produce *M. gallisepticum katE* transformants, electrocompetent *M. gallisepticum* cells were transformed with pOO55 and pOO56 as previously described [30]. Transformants were plated on SP-4 plates containing 4 µg mL^−1^ tetracycline for selection. Transformants were subjected to three rounds of filter cloning and more than one transformant from each series (named 55A–C and 56 A and C) was analyzed to ensure that results observed were not due to polar effects from the chromosomal transposon insertion site.

### H_2_O_2_ assay

Methods were adapted from Hames *et al.* [14]. Fifty-mL cultures of mycoplasmas were grown to mid-log phase. Cells were collected by centrifugation at 20,000×*g* for 20 min and washed three times with cold buffer containing 67.6 mM HEPES, 140 mM NaCl, and 7 mM MgCl_2_. Following resuspension to an OD_550_ of 1.0, 1-mL aliquots were placed in microcentrifuge tubes and incubated at 37°C for 1 h. 100 µM glycerol was added to tubes and samples were incubated at 37°C for an additional 2 h. H_2_O_2_ levels were measured using colorimetric test strips. All analyses were performed in quadruplicate. Median and median absolute deviations values were calculated. Statistical significance of results was calculated using one-way ANOVA and Tukey' *post hoc* test. Results represent three biological replicates with at least two technical replicates each.

### Preparation of cell extracts

Fifty-mL cultures of mycoplasma cells were collected by centrifugation at 20,000×*g* for 20 min and washed three times with cold PBS. The resulting cell pellet was resuspended in 1 mL cold PBS containing 1% sodium dodecyl sulfate and incubated at 37°C for 30 min. Protein concentration in cell lysates was determined using BCA assays (Pierce).

### Western blot analysis

Five µg of cell lysates was separated on a 10% sodium dodecyl sulfate-polyacrylamide gel. Following electrophoresis, proteins were transferred to a nitrocellulose membrane (GE) overnight at 100 mAmp. Membrane was probed with anti-6XHis primary antibodies (Immunology Consultants Laboratory, Inc.) at a dilution of 1∶1,000 followed by anti-rabbit immunoglobulin G-alkaline phosphatase secondary antibodies (Promega) at a dilution of 1∶7,500. Bands were visualized with 5-bromo-4-chloro-3-indolyl phosphate and nitro-blue tetrazolium.

### PCR analysis

Primers were designed to encompass *M. gallisepticum glpF, glpO,* and *glpK* to confirm absence of transposon insertion at this locus. Primers (5′-TCAAGTTCTGCTAGTAGCGG-3′ and 5′-AATGTATCAGATCACGCACC-3′) were synthesized (Fisher Scientific) and used with *Taq* polymerase for PCR with chromosomal DNA. PCR products were subjected to agarose gel electrophoresis and transformants were compared to *M. gallisepticum* R_low_.

### Caenorhabditis elegans growth conditions

All assays were performed with *C. elegans* N2 (Bristol). Nematodes were cultivated using standard practices [31]. Briefly, worms were cultivated on Nematode Growth Media (NGM) plates seeded with *E. coli* strain OP50 as a food source at room temperature on the bench top.

### *C. elegans* killing assays

Plates containing many large, gravid nematodes were bleached to obtain sterile eggs using standard procedures [31]. Eggs were hatched overnight in 10 mL of M9 buffer to obtain L1 larvae. Following incubation with 1 mM glycerol for 5–6 h, L1 larvae were washed with and resuspended in M9 buffer, and approximately 20–40 larvae were aliquotted into 24-well plate wells. The number of live worms per well (indicated by the presence of movement) was counted prior to adding any additional sample. Plates were incubated at room temperature on the bench top for 24 h and live worms were counted to measure survival. Worms were considered dead if they showed no movement in response to shaking of the plate.

To determine the susceptibility of worms to H_2_O_2_ using our assay conditions, L1 larvae were incubated with 1 mL of buffer containing 67.6 mM HEPES, 140 mM NaCl, and 7 mM MgCl_2_ and various concentrations of H_2_O_2_. Survival was measured at 24 h as described above. Statistical significance of results was calculated using one-way ANOVA. Results represent three biological replicates with at least two technical replicates each.

Mycoplasma samples were processed as described for hydrogen peroxide assays. Following initial 1-h incubation at 37°C, 1-mL samples were added to 24-well plate wells containing counted nematodes and glycerol. Worm survival was measured after 24 h incubation at room temperature. Conditions were optimized for maximum H_2_O_2_ production at room temperature by varying the OD_550_ of *M. gallisepticum* R_low_ and concentrations of glycerol. The concentration of H_2_O_2_ under these optimized conditions was established at 2-h intervals for each strain using the colorimetric test strips. Final nematode killing assays were performed with mycoplasmas at an OD_550_ of 0.75 in the presence or absence of 1 mM glycerol. Statistical significance of results was calculated using one-way ANOVA with Tukey' *post hoc* test and unpaired Student's T-test. Results represent three biological replicates with at least two technical replicates each.

## Results

### Analysis of *M. iowae* serovar K for catalase activity

We obtained a draft genome sequence of *M. iowae* serovar K, isolated from a turkey embryo [32]. The genomes of serovars K and I, which was isolated from a turkey air sac [32], are well conserved, with >99% nucleotide sequence conservation in most genes, and more substantive differences in genes encoding predicted lipoproteins and some hypothetical proteins. Differences in gene content are almost completely restricted to restriction systems, transposase fragments, and lipoprotein paralogs, plus the deletion in serovar K of two putative glycoconjugate synthesis genes.

**Figure 1:**
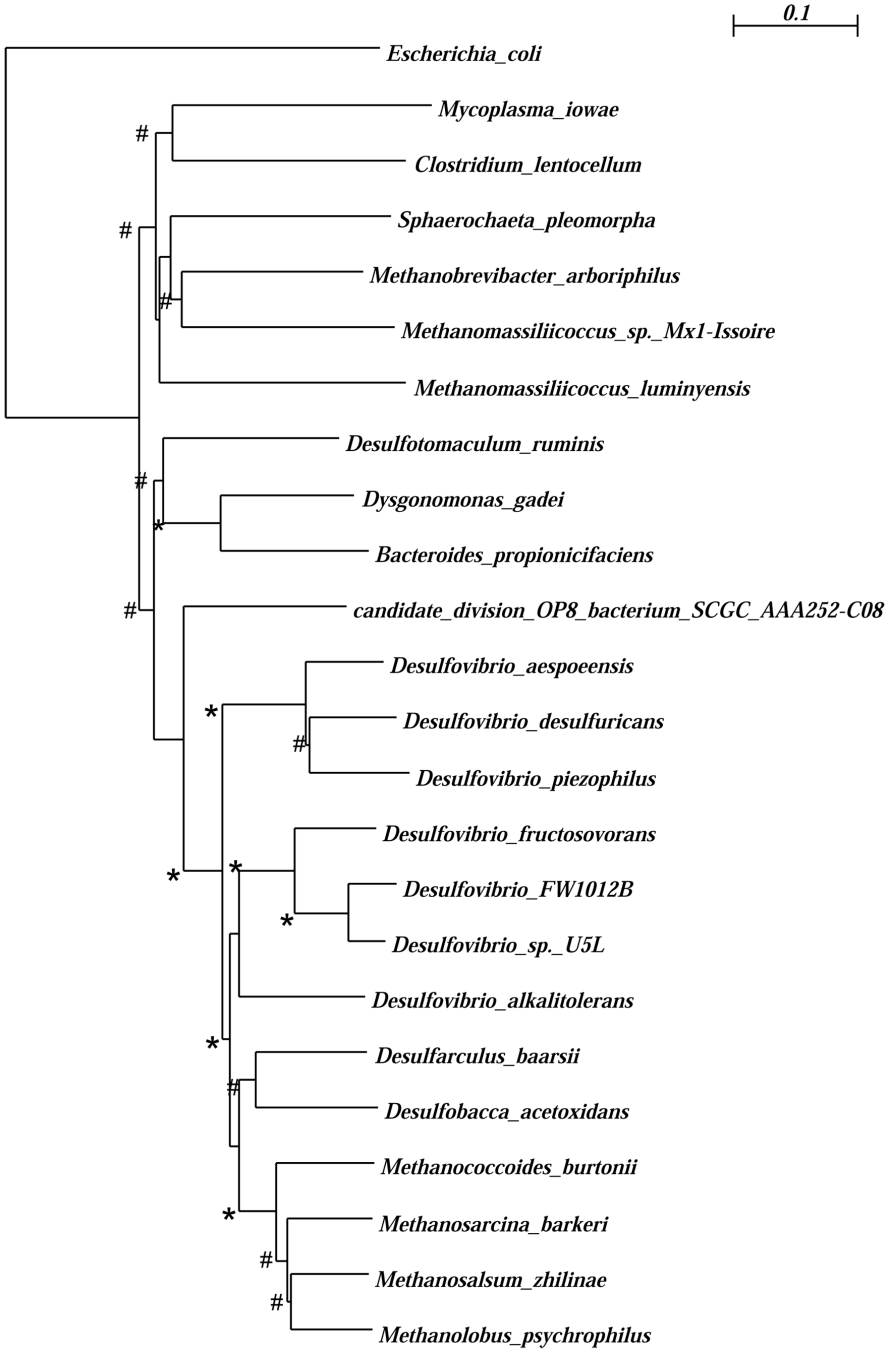
Phylogenetic tree of *M. iowae* serovar K catalase and close relatives, with *Escherichia coli katE* as an outgroup. All amino acid sequences included have an e value reported as 0 by PSI-BLAST with respect to *M. iowae* catalase. Scale bar, 0.05 substitutions per site. Bootstrap values out of 1000 trials are reported at nodes.

*glpF*, *glpK*, and *glpO* were present, constituting a complete pathway for assimilation of free glycerol and H_2_O_2_ production. However, *glpU* and *glpQ*, which import and break down host glycerophospholipids to glycerol-3-phosphate, the substrate for GlpO [33], [34], were absent. These findings suggest that *M. iowae* cannot use host cell phospholipids as a source of glycerol, leaving free glycerol, perhaps derived from host cells and/or other microflora, as the only source of substrate for GlpO. Although the genome had a typical complement of mycoplasmal genes associated with antioxidant functions, including genes encoding lipid hydroperoxide peroxidase, glutathione peroxidase, thioredoxins and thioredoxin reductases, flavodoxin and flavodoxin reductase, peroxiredoxin, and an OsmC-like protein possibly functioning in organic hydroperoxide reduction, two other genes stood out. One was a gene for superoxide dismutase, which among mycoplasmas has been found only in *Mycoplasma haemofelis* and *Mycoplasma haemocanis* [35], [36]. The other was a gene for catalase, which we call *katE*. It is present in both serovar K and the previously published serovar I [11], and the nucleotide sequence is nearly identical both in the coding region and the 434-bp non-coding region upstream. The predicted amino acid sequence for *M. iowae* serovar K catalase had 61% sequence identity to a homolog in the archaeon *Methanobrevibacter arboriphilus*, whose heme-dependent catalase activity has been characterized [37]. The predicted catalase protein contains all conserved amino acids of clade 3 heme-binding catalases as indicated on the Conserved Domain Database (CDD) [38], a group which it matches with an e-value of less than 10^-100^_._ Its closest relatives are all found in anaerobic prokaryotes (Fig. 1), among which *Clostridium lentocellum* and *Desulfotomaculum ruminis* are the only members of the closely related *Firmicutes* lineage. The lack of a close relationship with catalases from most *Firmicutes* is more consistent with *M. iowae* having acquired *katE* by horizontal gene transfer from an anaerobe than having retained it during evolution from a common ancestor with Gram-positive bacteria. Sequence alignment reveals conservation of predicted secondary structural elements and active site residues (Fig. S1).

**Figure 2:**
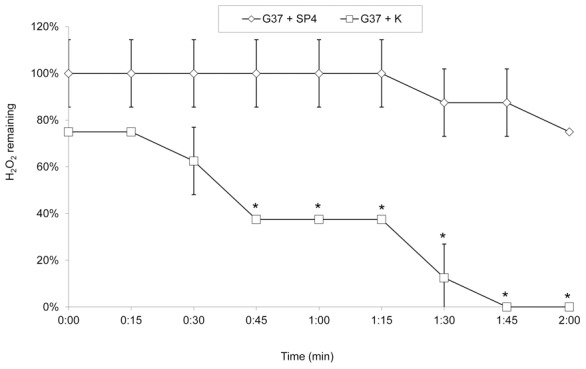
Degradation of H_2_O_2_ produced by an exogenous source of by *M. iowae*. H_2_O_2_ present in a mid-log phase culture of *M. genitalium* G37 was measured following the addition of an equal amount of SP-4 media (◊) or mid-log phase *M. iowae* serovar K culture (□). Error bars, SD. *, *p*<0.05 compared to control as determined by Student's T-test.

The presence of *katE* in *M. iowae* constitutes the first published account of a catalase gene in any mycoplasma species to date. Catalase activity was assessed in whole cells of *M. iowae* serovar K and *M. gallisepticum* strain R_low_, which has no catalase gene. Upon the addition of 3% H_2_O_2_, *M. iowae* produced robust bubbling which was absent from *M. gallisepticum* (Table 1), consistent with the presence of catalase activity in only *M. iowae*. Furthermore, *M. iowae* was capable of breaking down H_2_O_2_ produced by *M. genitalium*, a species previously shown to be a robust H_2_O_2_-producer [39]. Cells of both species were grown to mid-log phase in media containing 3% glycerol, which stimulates H_2_O_2_ production [19], [39]. Upon reaching mid-log phase, the cultures were combined and H_2_O_2_ concentration was monitored over time (Fig. 2). When an equal volume of *M. iowae* culture was added to the *M. genitalium* culture, the H_2_O_2_ concentration fell below detectable levels within 2 min. This decrease in the amount of H_2_O_2_ was not observed when warm SP-4 broth was added to *M. genitalium*. A repeated measures ANOVA revealed a significant difference between these two groups (*F*(1,8)  = 56.783, *p*<0.05), and significant differences were observed from 45 sec onward during the assay as determined by unpaired Student's T-test (*p*<0.05). These data are consistent with catalase activity by *M. iowae*.

**Table 1:** Catalase activity and H_2_O_2_ production by *M. iowae* serovar K, *M. gallisepticum* R_low_, and *M. gallisepticum* 55 and 56 series transformants containing *M. iowae katE*.

Strain	Catalase activity	H_2_O_2_ production (mg L^−1^)	
		100 µM glucose	100 µM glycerol
*M. iowae* serovar K	+	0±0*	0±0*
*M. gallisepticum* R_low_	−	1.6±0.1	3.8±0.3
55A	+	0±0*	0±0*
55B	+	0±0*	0±0*
55C	+	0±0*	0.1±0.1*
56A	+	0±0*	0±0*
56C	+	0.3±0.3*	0.6±0.4*

*P<0.05 compared to *M. gallisepticum* R_low_ H_2_O_2_ production with respective carbohydrate.

### Expression of *M. iowae katE* in a hydrogen peroxide-producing mycoplasma

**Figure 3:**
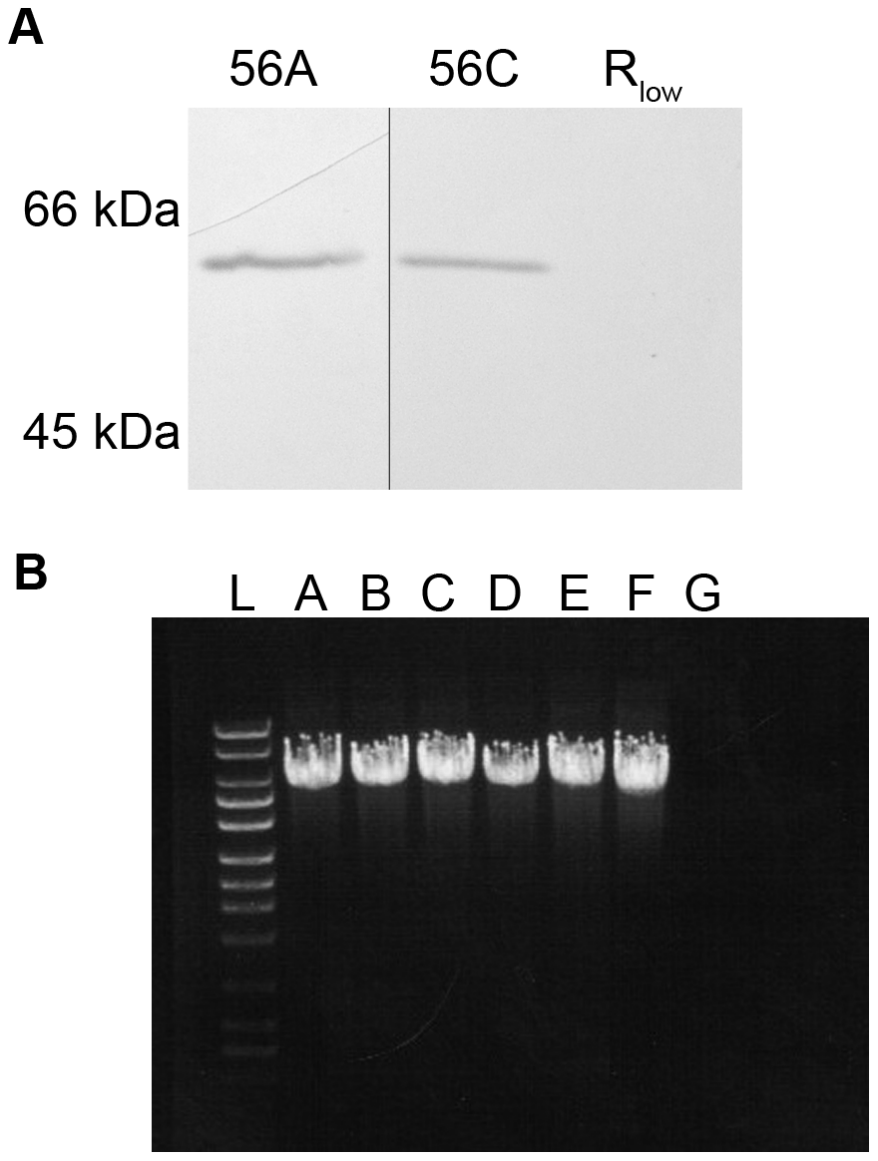
Production of catalase and integrity of glycerol metabolism operon in *M. gallisepticum* transformants. (a) Immunoblot of *M. gallisepticum* transformants 56A and 56C and *M. gallisepticum* R_low_ with anti-6X-His antibodies. (b) PCR products using primers for the putative *glpFOK* operon of *M. gallisepticum*. L, ladder with bands at 10, 8, 6, 5, 4, 3, 2.5, 2, 1.5, 1, 0.7, and 0.5 kb; A, *M. gallisepticum* R_low_; B–D, *M. gallisepticum* transformants 55A–C; E–F, *M. gallisepticum* transformants 56A and C; G, *M. iowae* serovar K.

To test the role of *katE* in the catalase activity of *M. iowae*, *M. gallisepticum* was transformed with the gene either without or with a C-terminal 6X-His tag to create transformant series 55 and 56, respectively [30]. This species was chosen as the recipient due to its ability to produce H_2_O_2_ and lack of other known toxins [40], [41]. We included the coding region of *katE* plus 239 nucleotides upstream of the start codon, anticipating that this region included a promoter, although no promoter sequences could be unambiguously identified by sequence inspection (not shown). Immunoblotting with anti-6X-His antibodies confirmed production by transformants of a protein migrating at approximately 60 kDa, the predicted size of *M. iowae* catalase, that was absent from untransformed *M. gallisepticum* (Fig. 3a). Addition of 3% H_2_O_2_ to all transformants resulted in bubbling, indicative of the presence of catalase activity (Table 1). Transformants were also analyzed for H_2_O_2_ production in the presence of 100 µM glucose or glycerol as previously described [14]. *M. iowae* produced no detectable H_2_O_2_ under any test conditions, including incubation with up to 3 mM glycerol (Table 1 and not shown). Although the parent strain produced robust H_2_O_2_ in the presence of 100 µM glycerol, all *katE* transformants displayed decreased H_2_O_2_ production, approaching or at undetectable levels. Individual transformants produced varying amounts of H_2_O_2_, but always less than the parent strain. There was a statistically significant difference between strains as determined by a one-way ANOVA (*F*(7,21)  = 73.102, *p*<0.0001 for 100 µM glucose and *F*(7,22)  = 96.076, *p*<0.0001 for 100 µM glycerol). A Tukey *post hoc* test revealed significant differences between *M. gallisepticum* and all catalase-containing strains, but no statistical differences between *M. iowae* and catalase-containing transformants following treatment with both carbohydrates, suggesting that *katE* is responsible for the catalase activity present in *M. iowae*. Because it was possible that introduction of the transposon carrying *katE* interfered with glycerol metabolism and therefore H_2_O_2_ production, PCR was performed on the *glpFOK* putative operon, which constitute the only genes known to be involved in glycerol metabolism in this organism. Wild-type *M. gallisepticum* and all transformants displayed a product of ∼5.7 kb (Fig. 3b). These results are consistent with reduction in H_2_O_2_ accumulation in transformants being due solely to catalase activity provided by *M. iowae katE*.

### H_2_O_2_-mediated toxicity in a *C. elegans* model

**Figure 4:**
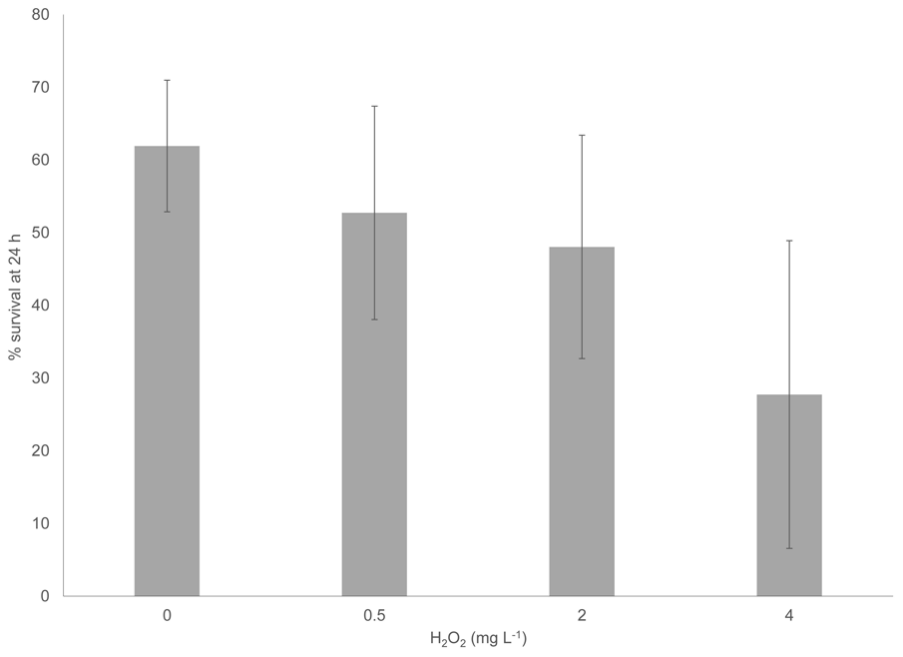
Survival of *C. elegans* upon exposure to increasing concentrations of H_2_O_2_. Error bars, SD.

**Figure 5:**
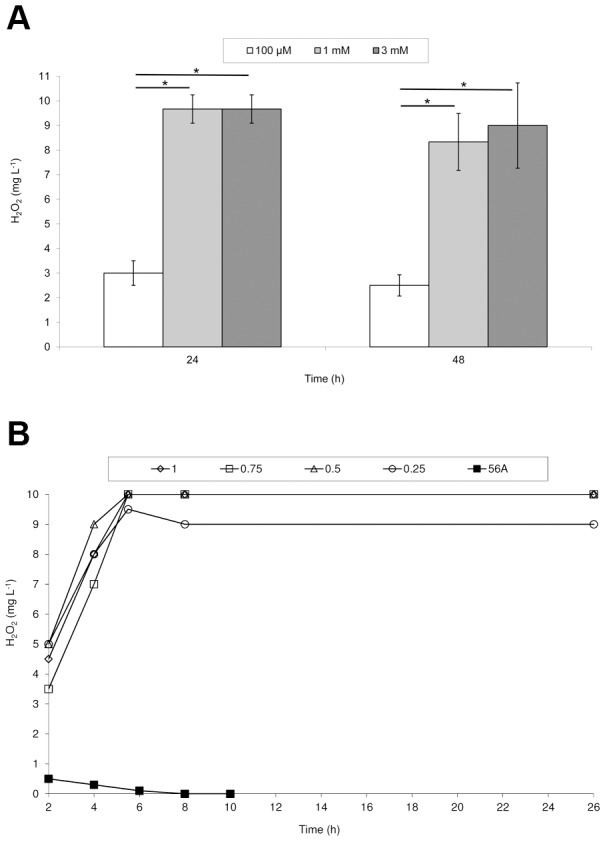
H_2_O_2_ accumulation by different amounts of *M. gallisepticum* with varying glycerol concentrations. (a) *M. gallisepticum* R_low_ at an OD_550_ of 1.0 with 100 µM (white bars), 1 mM (light grey bars), or 3 mM (dark grey bars) glycerol. Experiments were performed in triplicate. *, *p*<0.05 as determined by one-way ANOVA and Tukey's *post hoc* test. (b) *M. gallisepticum* R_low_ at an OD_550_ of 1 (◊), 0.75 (□), 0.5 (Δ), or 0.25 (x) with 1 mM glycerol and *M. gallisepticum* catalase-producing transformant 56A at an OD_550_ of 0.75 with 1 mM glycerol. Results are from one representative experiment.

Multiple studies have demonstrated the susceptibility of *C. elegans* to H_2_O_2_ [42], [43], but a *C. elegans* model for toxicity of H_2_O_2_ from mycoplasmas has never been reported. When *C. elegans* L1 larvae were incubated with increasing concentrations of H_2_O_2_ at room temperature, a significant decrease in survival at 24 h was evident as indicated by a one-way ANOVA (*F*(3,26)  = 5.911, *p* = 0.003) (Fig. 4). To maximize H_2_O_2_ output by *M. gallisepticum* after 24 h, thereby allowing for the most easily discernible results in subsequent assays, various concentrations of glycerol and *M. gallisepticum* cells were analyzed at room temperature to mimic conditions necessary for *C. elegans* toxicity assays (Fig. 5). H_2_O_2_ production by wild-type *M. gallisepticum* reached a distinct maximum level when the glycerol concentration was increased to 1 mM (Fig. 5a). There was a statistically significant difference between different glycerol concentrations at the same time point as determined by a one-way ANOVA (*F*(7,6)  = 124.842, *p*<0.0001 at 24 hr and *F*(7,6)  = 20.591, *p*<0.0001 at 48 hr). A Tukey post hoc test revealed a significant difference associated with treatment with 100 µM glycerol but not with higher concentrations. No statistically significant differences were observed for any glycerol concentrations with an increase in incubation time from 24 to 48 hrs. OD_550_ values ranging from 0.5 to 1.0 yielded the same level of H_2_O_2_ (Fig. 5b); therefore, we chose to use cells at an OD_550_ of 0.75 in the *C. elegans* assays. When transformant 56A, representing the *katE* transformants, was analyzed for H_2_O_2_ production with cells at an OD_550_ of 0.75 in the presence of 1 mM glycerol at room temperature, H_2_O_2_ was detectable, but present at more than an order of magnitude lower than wild-type *M. gallisepticum*, with H_2_O_2_ production peaking early and becoming undetectable after 10 h (Fig. 5b). Thus, the amount of H_2_O_2_ produced by both wild-type *M. gallisepticum* and transformant 56A under the conditions used for the *C. elegans* studies is markedly greater than those originally obtained with 100 µM glycerol over 2 h of incubation at 37°C.

**Figure 6:**
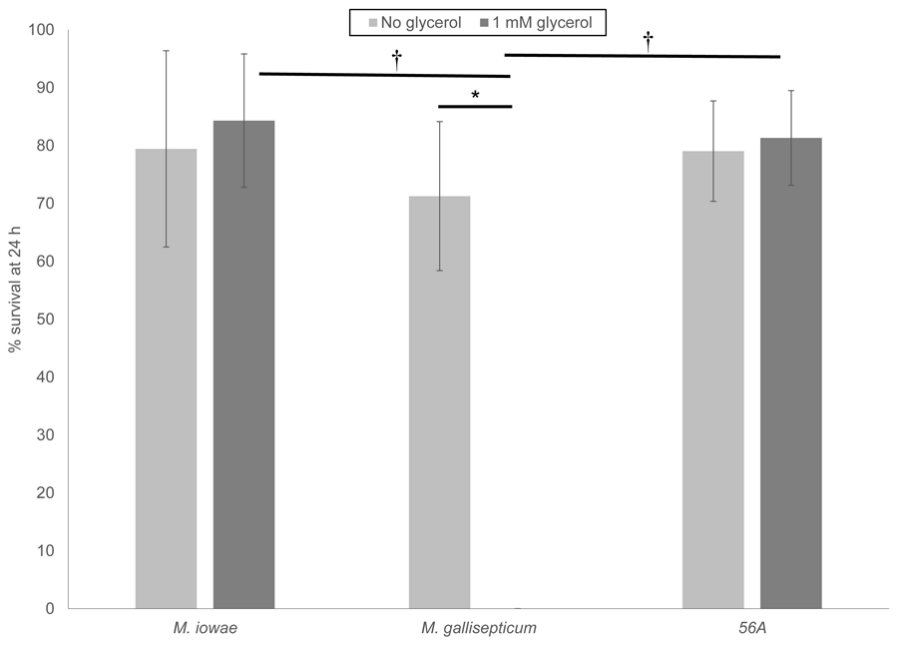
Toxicity of mycoplasmas to *C. elegans.* Toxicity was measured in the absence (light grey bars) and presence (dark grey bars) of 1 mM glycerol. Error bars, SD. *, *p*<0.05 as determined by unpaired Student's T-test. † *p*<0.05 as determine by one-way ANOVA and Tukey's *post hoc* test.

To compare H_2_O_2_ toxicity in the presence and absence of catalase-containing mycoplasmas, pre-counted *C. elegans* L1 larvae were incubated with 1 mL of *M. iowae*, *M. gallisepticum*, and *M. gallisepticum* transformant 56A with catalase activity at an OD_550_ of 0.75 in the presence or absence of 1 mM glycerol at room temperature, and survival was measured at 24 h (Fig. 6). In the absence of glycerol, leaving H_2_O_2_ production absent or minimal (see Table 1), incubation with all strains resulted in 60–80% survival of *C. elegans*, which were statistically similar as determined by a one-way ANOVA (*F*(2,21)  = 0.962, *p* = 0.398). Statistically significant differences were found between experiments with and without glycerol for *M. gallisepticum*, but not for *M. iowae* or transformant 56A as determined by Student's T-test. Wild-type *M. gallisepticum* allowed no survival of *C. elegans* at 24 h due to the high level of H_2_O_2_ production. Transformant 56A allowed increased survival of *C. elegans* as compared to wild-type *M. gallisepticum*, paralleling differences observed in H_2_O_2_ production under assay conditions (Fig. 5b). Catalase-producing *M. iowae* and transformant 56A were both statistically significantly different from untransformed *M. gallisepticum* in the presence of glycerol as determined by a one-way ANOVA (*F*(2,21)  = 292.531, *p*<0.001) and Tukey's *post hoc* test.

## Discussion

Despite the presence in *M. iowae* of an entire set of genes sufficient for importing and using free glycerol as a metabolite, this organism produced no detectable H_2_O_2_ under any conditions tested. We speculated that the absence of H_2_O_2_ accumulation was due to the presence and activity of a catalase, likely intracellular, which provides *M. iowae* with the ability to break down exogenous H_2_O_2_ and might remove H_2_O_2_ generated through the glycerol catabolism pathway before it accumulates to detectable levels. Consistent with this hypothesis, *M. gallisepticum* displayed catalase activity upon transformation with *M. iowae katE*. Significantly, catalase activity resulted in reduced or even undetectable H_2_O_2_ production by *M. gallisepticum* transformants, and a *C. elegans* toxicity assay revealed a correlation between reduced H_2_O_2_ production and decreased toxicity. A possible explanation for the absence of H_2_O_2_ produced by *M. iowae* in the presence of glycerol is that the GlpFKO pathway experiences low flux and produces only a small amount of H_2_O_2_. A second possibility is that *M. iowae* has a defect in its GlpFKO pathway resulting in the inability to utilize glycerol as a metabolite and therefore an absence of H_2_O_2_ production. Finally, the catalase activity may simply be so great as to prevent any accumulation of H_2_O_2_ generated by glycerol catabolism. Although the presence of these genes suggests that there is a use for these proteins in normal cellular function, further testing is needed to determine their expression, activity, and regulation. There might, for example, be conditions under which either catalase activity is reduced or glycerol catabolism is increased. Preliminary data suggest that a close relative of *M. iowae*, *M. penetrans*, produces minimal H_2_O_2_ amounts from glycerol catabolism (J. T. Newman and M. F. B., unpublished data), raising the possibility that this pathway is not very productive in this clade of mycoplasmas. Development of genetic tools for *M. iowae*, which have not been described, would be valuable for directly addressing the question of H_2_O_2_ production.

Our finding of reduced or no H_2_O_2_ production in the presence of catalase suggests a fundamental incompatibility between the presence of catalase and the use of H_2_O_2_ as a virulence factor by mycoplasmas, and provides insight into the correlation between the presence of glycerol catabolism genes and the use of H_2_O_2_ for virulence. If H_2_O_2_ is a widespread virulence factor among mycoplasmas, then this incompatibility is likely to explain the general absence of catalase from other mycoplasmas. The findings of this report support the idea that, although glycerol catabolism genes are widespread amongst mycoplasmas, their presence does not necessarily signal the use of H_2_O_2_ for virulence. In addition to its function in utilizing glycerol as an energy source, recent work has illustrated the importance of this pathway in other biochemical functions, such as the production of lipids [44].

*C. elegans* has previously been used to assay toxicity of several bacterial species via production of toxic molecules and infectious processes. The susceptibility of *C. elegans* to H_2_O_2_ has been well established and H_2_O_2_-mediated toxicity assays on solid media and in liquid have been used to study virulence caused via this mechanism [42], [43], although this is the first report of a *C. elegans* assay addressing mycoplasma toxicity. There are several benefits of using this method as opposed to standard tissue culture models. First, *C. elegans* is a more complex model than tissue culture cells, possessing several of the same innate immune defense mechanisms as more complex organisms [45], although whether this feature is important in this particular study is not clear. Second, previous studies have established a time frame of 2–5 d until bacteria can adequately colonize the gut of *C. elegans* and therefore cause damage via an infectious process [43]. Any death observed in the first 48 h is therefore due to diffusible molecules like H_2_O_2_. Third, tissue culture models of virulence require infection for long periods of time, and they cannot distinguish damage caused by H_2_O_2_, which occurs rapidly, and damage caused by toxins, which occurs more slowly, without the use of mutants, which can be difficult at minimum to construct using currently available molecular techniques [14]. Although there are limitations associated with comparing results obtained from this method to those observed in the natural host, such as examining the impact of H_2_O_2_ produced from exogenous or host-derived carbohydrates, it serves as a good system for examination of strains deficient in H_2_O_2_ production, such as the catalase-positive strains examined in this study.

One potential concern about the use of *C. elegans* as a reporter of H_2_O_2_-mediated toxicity is the question of whether binding of mycoplasmas to *C. elegans* impacts toxicity during the time frame required for these assays. Whether *M. iowae* binds to *C. elegans* under our experimental conditions, or under any conditions, is not known. Although *M. gallisepticum* can bind red blood cells at room temperature within 5 min [46], other pathogens with cell-associated toxins also bind externally to *C. elegans* within 2 d yet still require 4 d before any decrease in worm survival is observed [47]. Therefore, even though mycoplasmas might or might not bind to *C. elegans* during the 24-h incubation required for the toxicity assay employed in this study, one can be certain that any decrease in survival during this time frame is due to the production of diffusible molecules such as H_2_O_2_ and not cell-associated toxins.

## Supporting Information

Figure S1 **Alignment of catalase proteins closely related to that of** ***M. iowae*** **.** The uppermost track plots alignment entropy, with hotter colors at more variable positions than colder ones. Beneath the entropy plot the sequence coordinates are given for *M. iowae* catalase. The secondary structure for KatA from *Enterococcus faecalis* (PDB ID, 1SI8; [48]), the most closely related catalase for which structural information is available, is displayed beneath the alignment plot. Active site residues are indicated with asterisks. Red alpha-helices and yellow beta-strands predicted by SOPMA [26] are indicated at the bottom.
(PDF)
