# Supplementary figures and images for: Reduction of Hydrogen Peroxide Accumulation and Toxicity by a Catalase from Mycoplasma iowae
Source: PLoS One. 2014 Aug 15;9(8):e105188. doi: 10.1371/journal.pone.0105188 (PMC4134286; doi:10.1371/journal.pone.0105188)

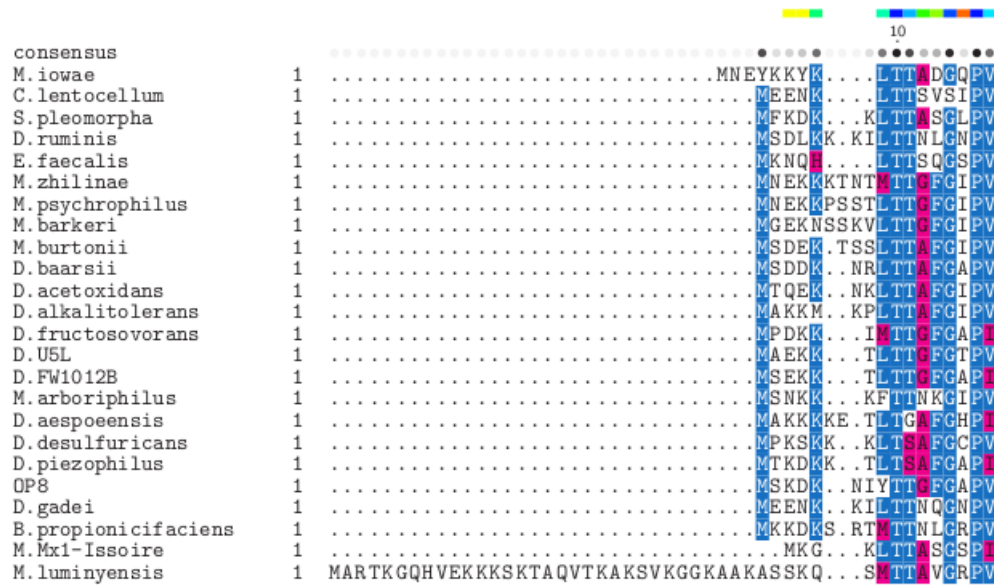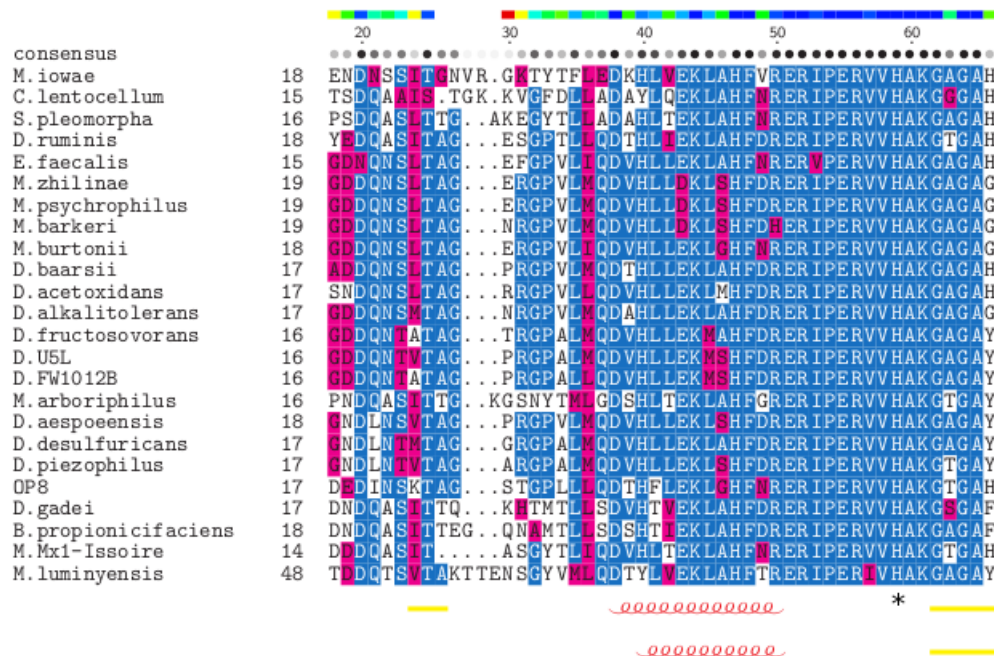

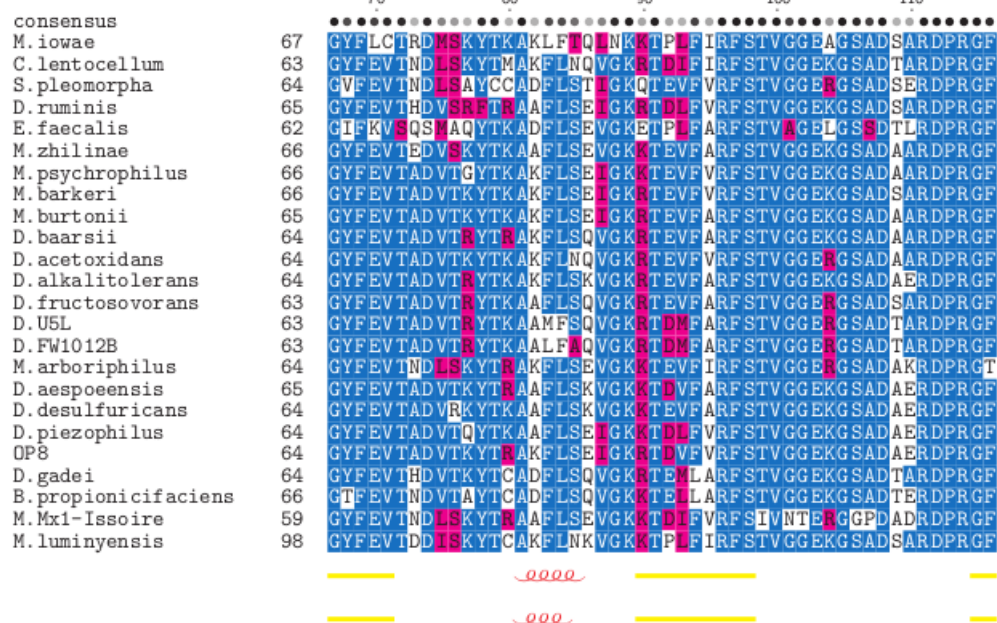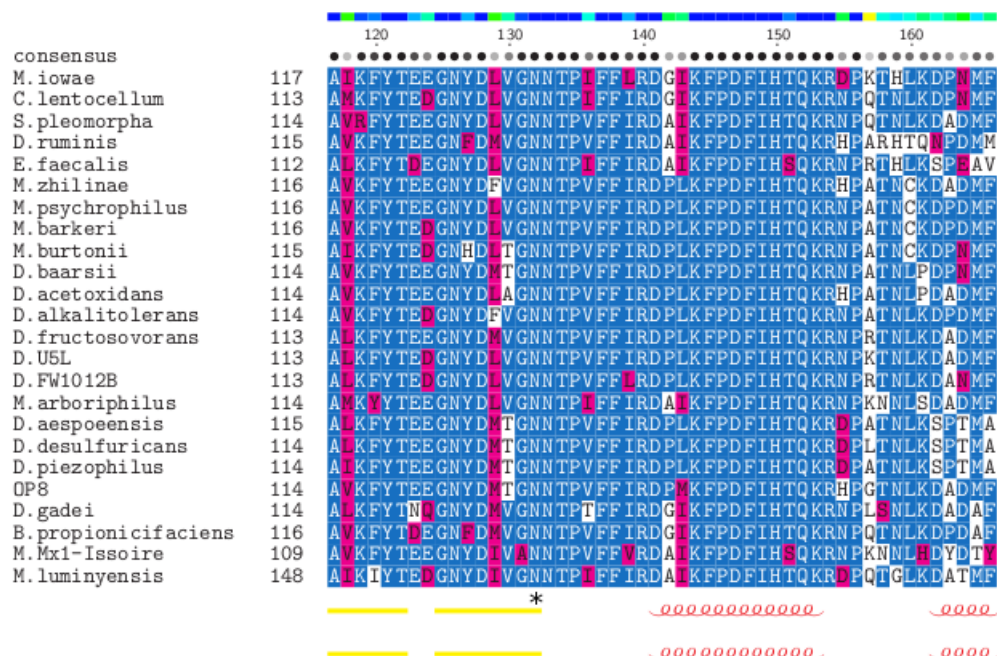

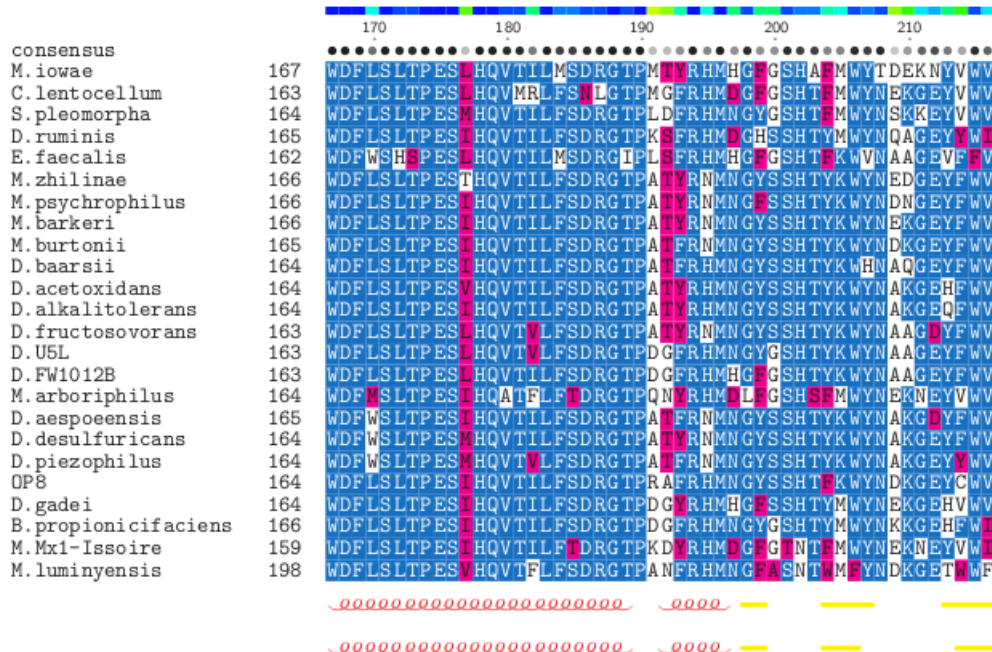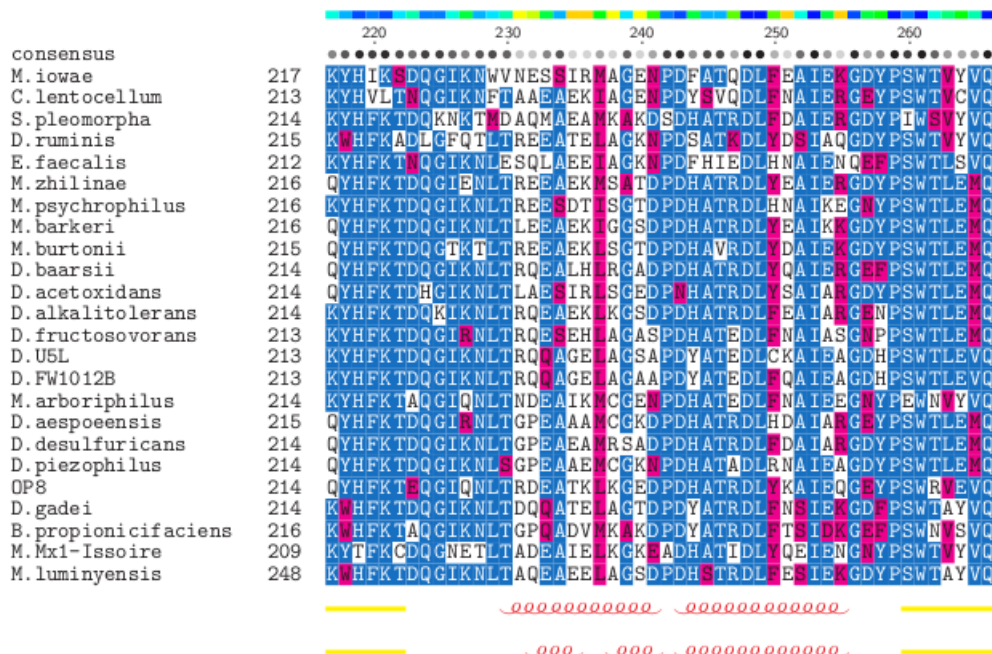

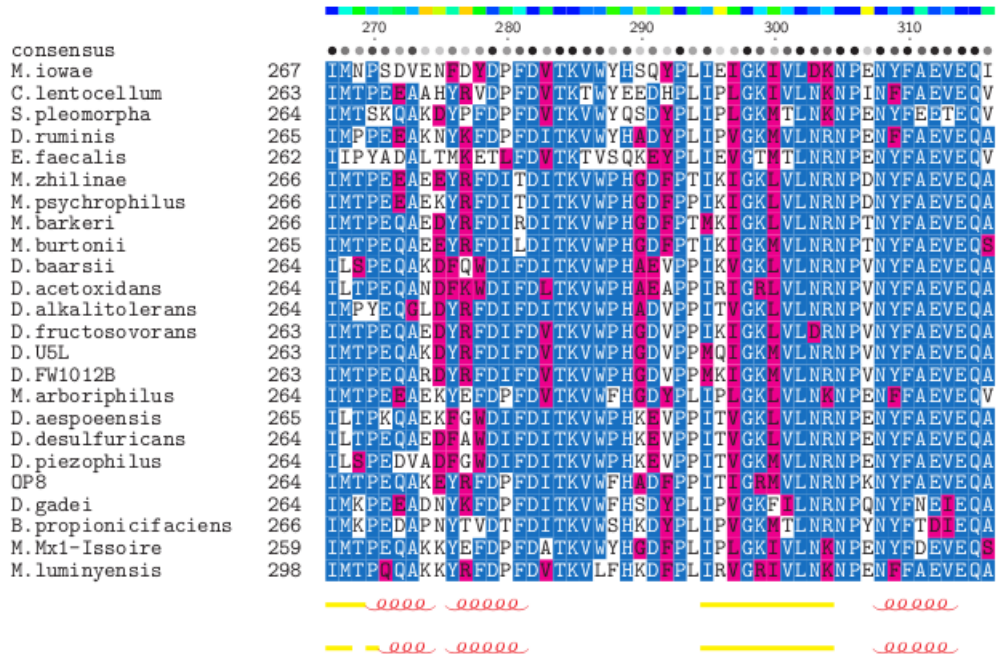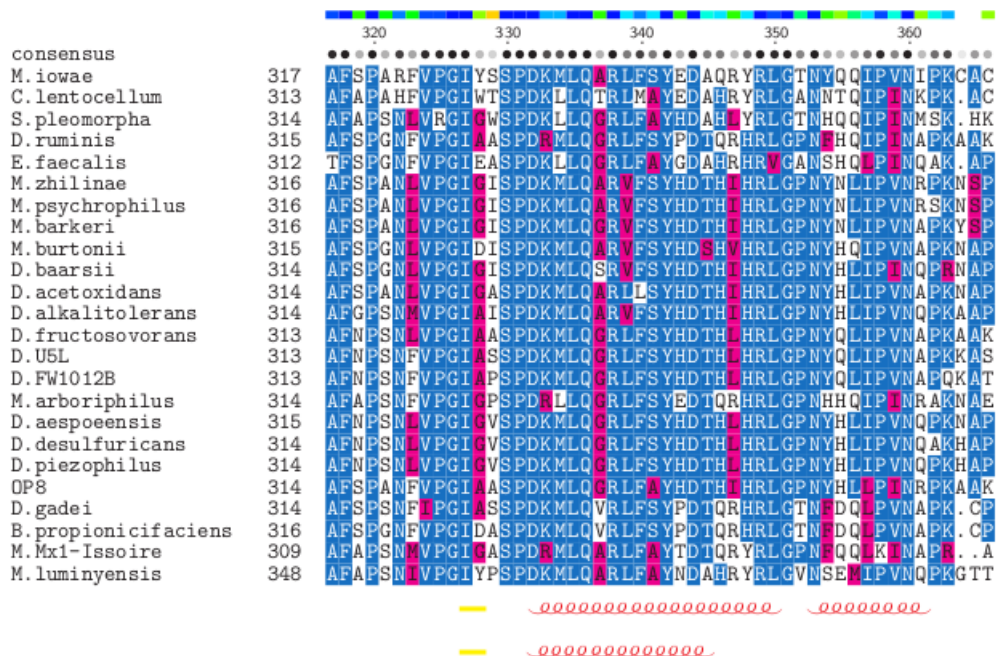

Supplement: Figure S1 — Alignment of catalase proteins closely related to that of M. iowae . The uppermost track plots alignment entropy, with hotter colors at more variable positions than colder ones. Beneath the entropy plot the sequence coordinates are given for M. iowae catalase. The secondary structure for KatA from Enterococcus faecalis (PDB ID, 1SI8; [48]), the most closely related catalase for which structural information is available, is displayed beneath the alignment plot. Active site residues are indicated with asterisks. Red alpha-helices and yellow beta-strands predicted by SOPMA [26] are indicated at the bottom. (PDF) [file pone.0105188.s001.pdf]
